# Machine learning-based remission prediction in rheumatoid arthritis patients treated with biologic disease-modifying anti-rheumatic drugs: findings from the Kuwait rheumatic disease registry

**DOI:** 10.3389/fdata.2024.1406365

**Published:** 2024-10-03

**Authors:** Ahmad R. Alsaber, Adeeba Al-Herz, Balqees Alawadhi, Iyad Abu Doush, Parul Setiya, Ahmad T. AL-Sultan, Khulood Saleh, Adel Al-Awadhi, Eman Hasan, Waleed Al-Kandari, Khalid Mokaddem, Aqeel A. Ghanem, Yousef Attia, Mohammed Hussain, Naser AlHadhood, Yaser Ali, Hoda Tarakmeh, Ghaydaa Aldabie, Amjad AlKadi, Hebah Alhajeri

**Affiliations:** ^1^College of Business and Economics, American University of Kuwait, Salmiya, Kuwait; ^2^Department of Rheumatology, Al-Amiri Hospital, Kuwait City, Kuwait; ^3^Department of Food and Nutritional Sciences, The Public Authority for Applied Education & Training, Shuwaikh Industrial, Kuwait; ^4^College of Engineering and Applied Sciences, American University of Kuwait, Salmiya, Kuwait; ^5^Computer Science Department, Yarmouk University, Irbid, Jordan; ^6^College of Agriculture, Govind Ballabh Pant University of Agriculture and Technology, Pantnagar, India; ^7^Department of Community Medicine and Behavioral Sciences, Kuwait University, Safat, Kuwait; ^8^Department of Rheumatology, Farwaniya Hospital, Kuwait City, Kuwait; ^9^Department of Rheumatology, Mubarak Al-Kabeer Hospital, Kuwait City, Kuwait; ^10^Department of Rheumatology, Al-Sabah Hospital, Kuwait City, Kuwait

**Keywords:** rheumatoid arthritis, bDMARDs, machine learning, explainable artificial intelligence, KRRD

## Abstract

**Background:**

Rheumatoid arthritis (RA) is a common condition treated with biological disease-modifying anti-rheumatic medicines (bDMARDs). However, many patients exhibit resistance, necessitating the use of machine learning models to predict remissions in patients treated with bDMARDs, thereby reducing healthcare costs and minimizing negative effects.

**Objective:**

The study aims to develop machine learning models using data from the Kuwait Registry for Rheumatic Diseases (KRRD) to identify clinical characteristics predictive of remission in RA patients treated with biologics.

**Methods:**

The study collected follow-up data from 1,968 patients treated with bDMARDs from four public hospitals in Kuwait from 2013 to 2022. Machine learning techniques like lasso, ridge, support vector machine, random forest, XGBoost, and Shapley additive explanation were used to predict remission at a 1-year follow-up.

**Results:**

The study used the Shapley plot in explainable Artificial Intelligence (XAI) to analyze the effects of predictors on remission prognosis across different types of bDMARDs. Top clinical features were identified for patients treated with bDMARDs, each associated with specific mean SHAP values. The findings highlight the importance of clinical assessments and specific treatments in shaping treatment outcomes.

**Conclusion:**

The proposed machine learning model system effectively identifies clinical features predicting remission in bDMARDs, potentially improving treatment efficacy in rheumatoid arthritis patients.

## 1 Introduction

Rheumatoid arthritis (RA) is a chronic inflammatory disease that can affect any number of joints and their surrounding synovial tissues. In fact, numerous clinical trials have demonstrated that between 30 and 40 percent of patients do not respond well to biologic therapy, and patient response rates continue to decline with each consecutive biologic (Keystone et al., 2004; Weinblatt et al., 1999). In addition, failure of treatment caused by ineffective biologics not only makes the individual feel more agony, but also drives up the cost of his medical care (Kievit et al., 2008).

In various clinical, genetic, and proteomic investigations, statistical techniques have been used to detect biomarkers that can anticipate the efficacy of biologics in patients diagnosed with rheumatoid arthritis (Park et al., 2015; Plant and Barton, 2020; Al-Herz et al., 2021). Machine learning approaches that supplement traditional statistical analysis may use this information to make reliable forecasts. In addition, machine learning can be generalized to a wider variety of types of data and is also capable of producing results in difficult scenarios (Guan et al., 2019; Norgeot et al., 2019; Alyasseri et al., 2022). The use of machine learning methodology is a viable option to investigate RA with varying clinical attributes. This enables the identification of critical clinical characteristics that are related to the desired outcomes and the ability to predict outcomes such as remission. The present investigation involved the development of machine learning (ML) models, including lasso regression, ridge regression, support vector machine (SVM), random forest, XGBoost, and Shapley additive explanation (SHAP), utilizing data from the Kuwait Registry for Rheumatic Diseases (KRRD) to identify clinical variables that predict remission in patients with rheumatoid arthritis (RA) who have undergone biologic treatment.

### 1.1 RA and machine learning

Numerous advances have been made in the field of machine learning that have led to the creation of techniques that can predict whether a patient will survive their condition (Lezcano-Valverde, 2017), experience disease activity (Ceccarelli, 2017), or do not respond to treatment (Norgeot et al., 2019). Compared to more traditional approaches, the predictive power of these models has been shown to be superior. In light of this, previous research has given us reason to believe that machine learning will be able to generate models with a higher degree of precision when it comes to identifying the target population for the early application of TNF inhibitors. This could potentially lead to more effective treatment plans and improved patient outcomes. However, more research is needed to fully evaluate the feasibility and practicality of implementing these models in clinical settings (Lee et al., 2020).

In a study conducted by Praestgaard and Iglesias-Rodriguez (2021), they used machine learning to determine a rule that could predict the response to sarilumab and differentiate between the responses to sarilumab and adalimumab, with a particular emphasis on blood biomarkers that may be used in clinical practice. They developed an algorithm that determined a straightforward and clinically applicable rule taking into account a huge number of combinatorial possibilities. Min and Haijiang (2020) created a machine learning technique to discover two-level and four-level RA classification experiments. These experiments investigated the impacts of the RA classification on different sizes of regions of interest. According to the study findings, integrated learning provides good classification impact and high precision for challenges involving small sample classifications.

### 1.2 Machine learning methods in RA

In this section, we will discuss the methods used in this paper to predict the most important characteristics for RA patients with KRRD.

#### 1.2.1 Support vector machine application in RA

A mathematical algorithm known as the support vector machine (SVM) is utilized for the examination of classification and regression. Compared to other classification methods, the SVM algorithm provides a number of distinct advantages. By using kernel functions to shift the data into a higher-dimensional space, it is resistant to overfitting and is able to manage nonlinearly separable data.

One of the many applications of SVM in RA is in the prediction of radiographic progression, which is an essential component of the prognosis of the disease. SVMs were used to construct a predictive model in a study by Kuo et al. (2018). This model was able to identify patients who were at high risk for radiographic progression.

The key hyperparameters in Support Vector Regression (SVR) are the penalty coefficient *C* and the kernel coefficient γ. The penalty coefficient *C* balances the trade-off between training error and model complexity, helping to avoid overfitting (Smola and Schölkopf, 2004). The kernel coefficient γ determines the influence range of a single training example, with low values indicating broader influence and high values indicating narrower influence (Schölkopf and Smola, 2002). SVR employs various kernel functions, such as linear, polynomial, radial basis function (RBF), and sigmoid, to model complex, non-linear relationships in the data (Cristianini and Shawe-Taylor, 2000). The SVR predictor function is:


$$f\left(x\right)=\overset{N}{\underset{i=1}{\sum }}\alpha_{i}K\left(x_{i},x\right)+b$$

where *K*(*x*_*i*_, *x*) is the kernel function, α_*i*_ are the Lagrange multipliers, and *b* is the bias term (Schölkopf, 1997).

The SVR optimization problem minimizes the following objective function:


$$\frac{1}{2}\overset{N}{\underset{i=1}{\sum }}\overset{N}{\underset{j=1}{\sum }}\alpha_{i}\alpha_{j}K\left(x_{i},x_{j}\right)-\overset{N}{\underset{i=1}{\sum }}\alpha_{i}+C\overset{N}{\underset{i=1}{\sum }}\max\left(0,|y_{i}-f\left(x_{i}\right)|-\epsilon \right)$$

subject to:


$$0\leq \alpha_{i}\leq C$$

where *y*_*i*_ are the actual values, *f*(*x*_*i*_) are the predicted values, and ϵ is a margin of tolerance where no penalty is given to errors (Vapnik, 1998).

#### 1.2.2 LASSO and ridge regression in RA

The primary objective of Lasso regression is to solve the problem of overfitting that occurs in linear regression models by introducing a penalty term that brings the coefficients of the regression model closer and closer to zero. This is accomplished by shrinking the coefficients of the regression model. The Lasso regression technique is particularly useful when dealing with high-dimensional datasets. It can effectively identify the most important features and exclude irrelevant ones from the model. The study conducted by Sun et al. (2022) employed LASSO regression to choose and construct a model for the variables of 14 clinical features and inflammatory indexes. The objective function that is minimized by the LASSO algorithm is expressed as Th Lasso loss function is given by:


(1) $$\begin{array}{l} L_{\text{lasso}}\left(\hat{\beta }\right)=\overset{n}{\underset{i=1}{\sum }}{\left(y_{i}-x_{i}^{\prime }\hat{\beta }\right)}^{2}+\lambda \overset{m}{\underset{j=1}{\sum }}|{\hat{\beta }}_{j}| \end{array}$$

where β represents the regression coefficients; *x* and *y* denote the inputs and the output, respectively. The variable *n* indicates the number of samples in the training dataset, and the hyper-parameter λ serves as the penalty parameter

The Ridge Regression (RR) technique, as proposed by Hoerl and Kennard (1970), is a predictive modeling approach that aims to stabilize regression estimates when independent variables are correlated. This technique effectively reduces the mean squared estimation error. In addition to identifying direct links, Ridge Regression also allows for the identification of multivariate relationships along a continuum by altering a tuning parameter. Morita et al. (2018) conducted a study in Japan aimed at developing a finger joint detection method for the automatic estimation of the progression of RA using ridge regression in Machine Learning. The results of the study indicate that the proposed method led to a significant improvement in the accuracy of finger joint detection. The ridge regression technique employs L2 regularization. The loss function in the ridge regression is defined as follows.


(2) $$\begin{array}{l} L_{\text{ridge}}\left(\hat{\beta }\right)=\overset{n}{\underset{i=1}{\sum }}{\left(y_{i}-x_{i}^{\prime }\hat{\beta }\right)}^{2}+\lambda \overset{m}{\underset{j=1}{\sum }}{\hat{\beta }}_{j}^{2}=||y-X\hat{\beta }|{|}^{2}+\lambda ||\hat{\beta }|{|}^{2} \end{array}$$

where *x* and *y* are the input and output vectors, respectively, *n* represents the number of samples in the training dataset, β denotes the regression coefficients, and λ is the penalty parameter.

#### 1.2.3 Random forest in predicting RA

The Random Forest algorithm is a commonly employed methodology within the domain of machine learning, which is used for the objectives of classification, regression, and feature selection. It is an ensemble learning approach that makes predictions using several decision trees. The Random Forest algorithm involves training individual decision trees in distinct subsets of both data and features. This improves the model's generalizability and reduces overfitting. The final forecast is then made using the aggregated predictions of all the independent decision trees. When the Random Forest (RF) algorithm processes an input vector (x) that includes the values of various evidential features analyzed for a particular training area, it generates a set of K regression trees and averages their results. Following the growth of *K* trees ${\left\{T\left(x\right)\right\}}_{1}^{k}$, the RF regression predictor can be expressed as the aggregation of the predictions from these trees (Breiman, 2001).


$${\hat{f}}_{\text{rf}}^{k}\left(x\right)=\frac{1}{k}\overset{k}{\underset{i=1}{\sum }}T\left(x_{i}\right)$$

Random Forest (RF) modeling in R utilizes the randomForest package, operating on the ensemble technique principle, which combines multiple classification and regression trees (CARTs) (Breiman and Cutler, 2004) to enhance accuracy and prevent overfitting. Two key parameters in ranom forest model are Mtry and Ntree (Belgiu and Dragut, 2016). Mtry determines the number of variables selected and split at each node, while Ntree specifies the number of trees grown. For regression, the Mtry value is generally the total number of observations divided by three. The data was tuned to identify the optimal Mtry, using the default 500 trees for model construction.

#### 1.2.4 XG Boost in predicting RA

The study used the XGBoost machine learning model to analyze the significant clinical characteristics associated with responses to bDMARD. This approach relied on the utilization of explainable artificial intelligence (XAI) (Arrieta et al., 2020).

The use of the XGBoost model has been widely used in various domains, including the prediction of the diagnosis of chronic kidney disease (Ogunleye and Wang, 2019). Furthermore, a separate study used the XGBoost model to accurately and promptly forecast the clinical response to methotrexate treatment in juvenile idiopathic arthritis (Mo et al., 2019). The research used the XGboost machine learning model to determine the primary factors and their impact on predicting remissions via the application of explainable artificial intelligence.

XGBoost is a machine learning model employed for time series forecasting, which utilizes an ensemble of decision trees (Dezhkam and Manzuri, 2023). The process of constructing these trees is directed by a gradient descent algorithm, aiming to reduce the loss function of the most recent tree (Yang and Shami, 2020).


$$L_{T}\left(F\left(x_{i}\right)\right)=\overset{N}{\underset{i=1}{\sum }}\chi \left(y_{i},F_{T}\left(x_{i}\right)\right)+\overset{T}{\underset{t=1}{\sum }}\Pi \left(f_{t}\right)$$

The function χ(·) represents a specified loss function that measures the differences between the predicted and actual target values. The term *F*_*T*_(*x*_*i*_) denotes the forecast for the *i*-th sample at the *T*-th iteration of boosting. The regularization term Π(*f*_*t*_) is given by:


$$\Pi \left(f\right)=\alpha K+\frac{1}{2}\lambda \overset{K}{\underset{j=1}{\sum }}w_{j}^{2}+\kappa$$

where *K* is the number of leaves in the model, α is the complexity parameter, λ is the L2 norm of the weight regularization, and κ is a constant coefficient. The regularization term Π(·) serves to penalize the complexity of the model, helping to prevent overfitting.

## 2 Methodolodgy

### 2.1 Patients data

All RA patients who participated in this investigation were required to have their information entered in the Kuwait Registry of Rheumatic Diseases between January 1, 2013, and December 30, 2022. (KRRD). The KRRD is a national registry for patients who belong to the age group 18 years and older, who suffer from rheumatic diseases. Patients diagnosed with RA who met the criteria established by the American College of Rheumatology (ACR) (Aletaha et al., 2010) and registered for the study between January 2013 and December 2022 were considered for participation in the research. The registry compiles information related to patients' demographic profiles, clinical features, disease activity, and responses to treatment. The purpose of the research is to analyze the long-term effectiveness of different treatments for RA patients registered in KRRD using machine learning algorithms.

In this study, clinical data was meticulously collected and recorded in the Kuwait Registry of Rheumatic Diseases (KRRD). The data collection process involved several layers of verification to ensure accuracy and reliability. Initially, clinical data, including patient demographics, disease characteristics, treatment regimens, and results, were recorded by treating rheumatologists and trained healthcare professionals during routine clinical visits.

To ensure data integrity, these entries were subsequently reviewed and cross-verified with the medical records of the patients. This process included confirming the diagnosis of rheumatoid arthritis (RA) based on established criteria, treatment details (specifically the use of biologic DMARDs) and clinical results. Regular audits were conducted to check for discrepancies or inconsistencies in the data.

Regarding the evaluation of the effects of each biologic DMARD, our analysis was carefully designed to isolate the impact of individual drugs. Patients included in the study were those who exclusively received a specific bDMARD without concomitant use of other biologics. This approach helped minimize confounding effects of polypharmacy and allowed a clearer assessment of each drug's efficacy and associated clinical outcomes. Moreover, to account for the influence of conventional synthetic DMARDs (csDMARDs), our analysis controlled their use, ensuring that the effects attributed to bDMARDs were as isolated as possible.

Information on RA was collected from patient visits to the rheumatology clinics of four of the most recognizable public hospitals in Kuwait. Due to the diverse population of the country, the hospitals chosen are spread over several governorates. This research was made possible by the approval of the KRRD by the Ethics Committees of both the Faculty of Medicine of Kuwait University and the Ministry of Health. In addition, all participating patients who signed up for the registry did so voluntarily and with their informed consent (Al-Herz et al., 2016). We searched public hospitals in Kuwait in search of patients to enroll in our study. Clinical data collected at enrollment allowed for prediction of remission (DAS-28 < 3.2) at 1-year follow-up. In this study, 1,968 patients with rheumatoid arthritis (RA) were included with a total of 11,195 follow-up visits.

#### 2.1.1 Calculating RA indices

The DAS28 and CDAI indices, also known as the “golden standards” for RA, are utilized to determine the degree to which RA disease activity is present (Salaffi et al., 2009; Muñoz et al., 2017). These involve the following: TJC28: The number of tender joints (0 − 28); SJC28: The number of swollen joints (0 − 28); D ESR: erythrocyte sedimentation rate (in mm/h); CRP: C-reactive protein (CRP) may be used instead of ESR in the calculation; and GH: Global health assessment of the patient (from 0 = best to 100 = worst) (see Equation 3).


$$\begin{array}{l} \text{DAS}28=0.56\times \sqrt{TJC28}+0.28\times \sqrt{SJC28}+0.70 \end{array}$$

(3) $$\begin{array}{l} \times \ln\;\left(ESR\;Or\;CRP\right)+0.014\times GH. \end{array}$$

#### 2.1.2 Shapley additive explanations approach

The SHAP approach (Shapley Additive Explanations) is a powerful method used to interpret and explain the results of machine learning models (Cravo et al., 2022; Chen, 2023; Pezoa et al., 2023; Lin et al., 2023; Obaido et al., 2022; ER, 2023; Chen, 2023; Obaido et al., 2022). It is based on game theory and provides a unified method for interpreting machine learning models (Chen, 2023). The SHAP method quantifies the contribution that each characteristic brings to the prediction made by the model (Chen, 2023). Machine learning algorithms, including feature selection and SHAP-based models, have been used to predict remission in patients with RA treated with biologics (Koo et al., 2021b; Alsaber, 2023). These models have identified important clinical characteristics such as age, rheumatoid factor, erythrocyte sedimentation rate, disease duration, and C-reactive protein, which help predict remission in response to different biologic treatments (Koo et al., 2021b; Alsaber, 2023). The SHAP method exhibits consistent feature importance irrespective of the model structure and the direction of effect of the predictive variables. This gives medical professionals insight into the achievement of remission and helps them identify potential factors that could impact their choice of bDMARDs. Furthermore, the SHAP approach shows a consistent importance of features regardless of the direction of the predictive variables (Koo et al., 2021a).

#### 2.1.3 Data processing

The data preprocessing involved several critical steps to ensure the quality and reliability of the modeling results. These steps included normalization, which was applied to appropriately scale the features, ensuring that all variables contributed equally to the model. Outliers were identified and removed to prevent them from skewing the results and adversely affecting the model performance. Therefore, in the initial step, the data were pre-processed to ensure the quality and reliability of the modeling results. All missing data were treated using the missForest approach (Dong et al., 2021; Alsaber et al., 2021, 2020).

#### 2.1.4 Statistical analysis procedures

Initially, patients were classified into two groups according to their disease activity score 28 (DAS-28). Those with a DAS-28 < 3.2 were placed in the low DAS-28 category, while patients with a DAS28 ≥ 3.2 were classified as having a high DAS-28 category. The statistical analysis procedure involved a comprehensive approach using various sets of data and parameters. The primary data set consisted of follow-up information from rheumatoid arthritis (RA) patients treated with biologic disease-modifying antirheumatic drugs (bDMARDs), including detailed clinical characteristics such as demographic factors, disease activity, and treatment responses. This study examined different types of biologic disease-modifying antirheumatic drugs (bDMARD) such as etanercept, adalimumab, golimumab, infliximab, abatacept, and tocilizumab and other treatment modalities among patients with rheumatoid arthritis (RA). The analysis included calculating the mean, standard deviation (SD), and percentage values for each data set. The *t* test and analysis of variance (ANOVA) were used to compare the means of two groups and the means of three or more groups, respectively. In addition, the Chi-square test was used to test the relationship between categorical variables. *p*-value < 0.05 was considered to examine the statistically significant difference. Moreover, machine learning models (SVM, Lasso, Ridge, RF, and Xgboost) were used to examine the significant predictors of RA. The performance of the machine learning models was evaluated based on their accuracy and the Area Under the Receiver Operating Characteristic (AUROC) curve. A model with perfect performance would have an AUC of 1, while a random guess would result in an AUC of 0.5.

The criteria used to evaluate model performance are sensitivity, specificity, precision, and accuracy. Statistical analyzes were performed with the R software version 3.6.1 (R Foundation for Statistical Computing, Vienna, Austria), and model training was performed with the R caret package and the SHAPforxg-boost package.

## 3 Results

### 3.1 Clinical characteristics of the patients

Table 1 demonstrates the clinical characteristics of 1,961 patients diagnosed with rheumatoid arthritis (RA) from the Kuwait Registry for Rheumatic Diseases (KRRD). Patients are divided into two groups according to their disease activity score 28 (DAS28) values: those with a DAS28 score < 3.2, indicating low disease activity, comprising 1,328 patients, and those with a DAS28 score ≥ 3.2, indicating moderate to high disease activity, comprising 633 patients.

**Table 1:** Clinical characteristics of the 1,961 RA patients from KRRD.

	**DAS28** < 3.2 (*N* = 1, 328)	**DAS28** ≥3.2 (*N* = 633)	**Total** (*N* = 1, 961)	*p* **value**
**Age, mean (SD), year**				<0.952^b^
Mean (SD)	56.0 (12.8)	56.1 (12.6)	56.0 (12.7)	
Range	17.6 − 94.4	20.3 − 86.3	17.6 − 94.4	
**Gender**				<0.001^a^
Female	816.0 (61.4%)	459.0 (72.5%)	1275.0 (65.0%)	
**Disease duration, mean (SD), year**				<0.750^b^
Mean (SD)	11.1 (6.7)	11.0 (7.6)	11.1 (7.0)	
Range	0.2 − 33.6	0.1 − 36.6	0.1 − 36.6	
**BMI**				0.161^b^
Mean (SD)	29.2 (6.1)	29.7 (6.6)	29.4 (6.3)	
Range	16.2 − 67.4	16.6 − 64.9	16.2 − 67.4	
**Current steroid**				<0.001^a^
Yes	112.0 (9.4%)	116.0 (19.8%)	228.0 (12.8%)	
**VAS**				<0.001^b^
Mean (SD)	0.8 (1.7)	4.0 (2.6)	1.8 (2.5)	
Range	0.0 − 10.0	0.0 − 10.0	0.0 − 10.0	
**ESR**				<0.001^b^
Mean (SD)	22.0 (19.0)	47.2 (24.5)	30.3 (24.1)	
Range	0.0 − 95.0	0.0 − 120.0	0.0 − 120.0	
**CRP**				<0.001^b^
Mean (SD)	4.3 (4.4)	6.8 (6.0)	5.0 (5.0)	
Range	0.0 − 21.0	0.0 − 21.0	0.0 − 21.0	
**DAS28**				<0.001^b^
Mean (SD)	2.0 (0.7)	4.3 (0.8)	2.8 (1.3)	
Range	0.0 − 3.2	3.2 − 7.8	0.0 − 7.8	
**SDAI**				<0.001^b^
Mean (SD)	6.0 (4.8)	13.7 (4.8)	7.2 (5.5)	
Range	0.0 − 21.0	2.0 − 21.0	0.0 − 21.0	
**CDAI**				<0.001^b^
Mean (SD)	2.1 (4.2)	17.1 (11.4)	6.9 (10.1)	
Range	0.0 − 33.0	0.0 − 73.0	0.0 − 73.0	
**HAQ**				<0.001^b^
Mean (SD)	0.9 (0.6)	1.1 (0.7)	1.0 (0.7)	
Range	0.1 − 3.0	0.1 − 3.0	0.1 − 3.0	
**Patient's global assessment**				<0.001^b^
Mean (SD)	0.8 (1.7)	3.9 (2.6)	1.8 (2.5)	
Range	0.0 − 10.0	0.0 − 10.0	0.0 − 10.0	
**Physician's global assessment**				<0.001^b^
Mean (SD)	0.5 (1.2)	2.9 (2.3)	1.3 (1.9)	
Range	0.0 − 10.0	0.0 − 10.0	0.0 − 10.0	
**RF**				0.001^a^
Positive	926.0 (75.5%)	478.0 (82.1%)	1, 404.0 (77.6%)	
**ANTI CCP**				0.182^a^
Positive	700.0 (66.2%)	344.0 (69.6%)	1, 044.0 (67.3%)	
**ANA**				<0.001^a^
Positive	294.0 (28.1%)	177.0 (37.0%)	471.0 (30.9%)	
**OnDMARD**				0.049^a^
Yes	1, 098.0 (82.7%)	500.0 (79.0%)	1, 598.0 (81.5%)	

^a^Pearson's Chi-squared test, ^b^Linear Model ANOVA/*t*-test.

From Table 1, the average age of the patients in both groups is around 56 years, with no significant differences between the two groups (*p* = 0.962). A significantly higher percentage of women are in the higher DAS28 category (72.5%) compared to the lower DAS28 category (61.4%) (*p* < 0.001). The duration of the disease appears to be quite similar for both groups, with an average of 11.1 years for the DAS28 < 3.2 group and 11.0 years for the DAS28 ≥ 3.2 group (*p* = 0.750). Furthermore, a significantly higher percentage of patients in the DAS28 ≥ 3.2 group (19.8%) are currently on steroids compared to the DAS28 < 3.2 group (9.4%) (*p* < 0.001). Patients in the DAS28 ≥ 3.2 group have significantly higher mean scores for VAS, ESR, CRP, DAS28, SDAI, CDAI, HAQ, patient's global assessment and physician's global assessment (*p* < 0.001 for all), indicating worse disease severity and increased inflammation. The percentage of patients with positive RF is significantly higher in the DAS28 ≥ 3.2 group (82.1%) compared to the DAS28 < 3.2 group (75.5%) (*p* = 0.001). Furthermore, the percentage of ANA positive patients is also significantly higher in the DAS28 ≥ 3.2 group (37.0%) compared to the DAS28 < 3.2 group (28.1%). There are no significant differences in the percentage of patients who are anti-CCP positive between the two groups (*p* = 0.178). The percentage of patients with DMARD is slightly, but significantly, higher in the DAS28 < 3.2 group (82.7%) compared to the DAS28 ≥ 3.2 group (79.0%) (*p* = 0.049).

Further, Table 2 provides a breakdown of the use of different types of biologic disease-modifying antirheumatic drugs (bDMARDs) and other treatment modalities among patients with rheumatoid arthritis (RA), as recorded in the Kuwait Registry for Rheumatic Diseases (KRRD). It categorizes patients into two groups based on their Disease Activity Score 28 (DAS28): a group with a DAS28 score less than 3.2, consisting of 8,284 patient visits, and another group with a DAS28 score ≥ 3.2, indicating moderate to high disease activity, consisting of 2,909 patient visits. From Table 2, it has been found that the use of Rituximab and Adalimumab is relatively similar between the two groups, with no statistically significant differences (*p* = 0.207 and *p* = 0.844, respectively). Tocilizumab use is significantly higher in the low disease activity group (DAS28 < 3.2), with 22.2% of these patients taking this medication compared to only 12.3% in the moderate to high disease activity group (DAS28 ≥ 3.2) (*p* < 0.001). Etanercept and Infliximab are also used more frequently in the low disease activity group (5.2% and 4.3% respectively) than in the moderate to high disease activity group (4.0% and 5.5% respectively), with these differences statistically significant (*p* = 0.009 and *p* = 0.007 respectively).

**Table 2:** Different types of biologic disease-modifying antirheumatic drugs (bDMARDs) and other treatment modalities among patients with rheumatoid arthritis (RA).

	**DAS28** < 3.2 (*N* = 8, 284)	**DAS28** ≥3.2 (*N* = 2, 909)	**Total** (*N* = 11, 193)	***p*** **value**
Rituximab	983.0 (11.9%)	371.0 (12.8%)	1, 354.0 (12.1%)	0.207^a^
Adalimumab	501.0 (6.0%)	173.0 (5.9%)	674.0 (6.0%)	0.844^a^
Tocilizumab	1, 835.0 (22.2%)	358.0 (12.3%)	2193.0 (19.6%)	<0.001^a^
Etanercept	427.0 (5.2%)	115.0 (4.0%)	542.0 (4.8%)	0.009^a^
Abatacept	522.0 (6.3%)	312.0 (10.7%)	834.0 (7.5%)	<0.001^a^
Infliximab	355.0 (4.3%)	160.0 (5.5%)	515.0 (4.6%)	0.007^a^
All bDMARDs	6, 602.0 (79.7%)	2, 368.0 (81.4%)	8, 970.0 (80.1%)	0.047^a^
Patients on biologics	4, 875.0 (58.8%)	1, 603.0 (55.1%)	6, 478.0 (57.9%)	<0.001^a^
**Treatment type**				0.298^a^
Combination	3, 283.0 (67.3%)	1, 102.0 (68.7%)	4, 385.0 (67.7%)	
Monotherapy	1, 592.0 (32.7%)	501.0 (31.3%)	2, 093.0 (32.3%)	

^a^Pearson's Chi-squared test.

In contrast, Abatacept is significantly more common in the moderate to high disease activity group (10.7%) than in the low disease activity group (6.3%) (*p* < 0.001). The general use of all bDMARDs is slightly higher in the moderate to high disease activity group (81.4%) compared to the low disease activity group (79.7%), and this difference is statistically significant (*p* = 0.047). The use of biological monotherapies or combination therapies (represented by the variables “Patients on biologics” and “Treatment type”) does not differ significantly between the two groups. Among those on biological therapies, the majority are on combination therapy in both groups (67.3% in the low disease activity group and 68.7% in the high disease activity group) with no significant differences between the two groups (*p* = 0.280).

### 3.2 Highlighting the top clinical features that influencing DAS28 using SHAP approach

#### 3.2.1 SHAP results for RA patients with cDMARD

**Figure 1:**
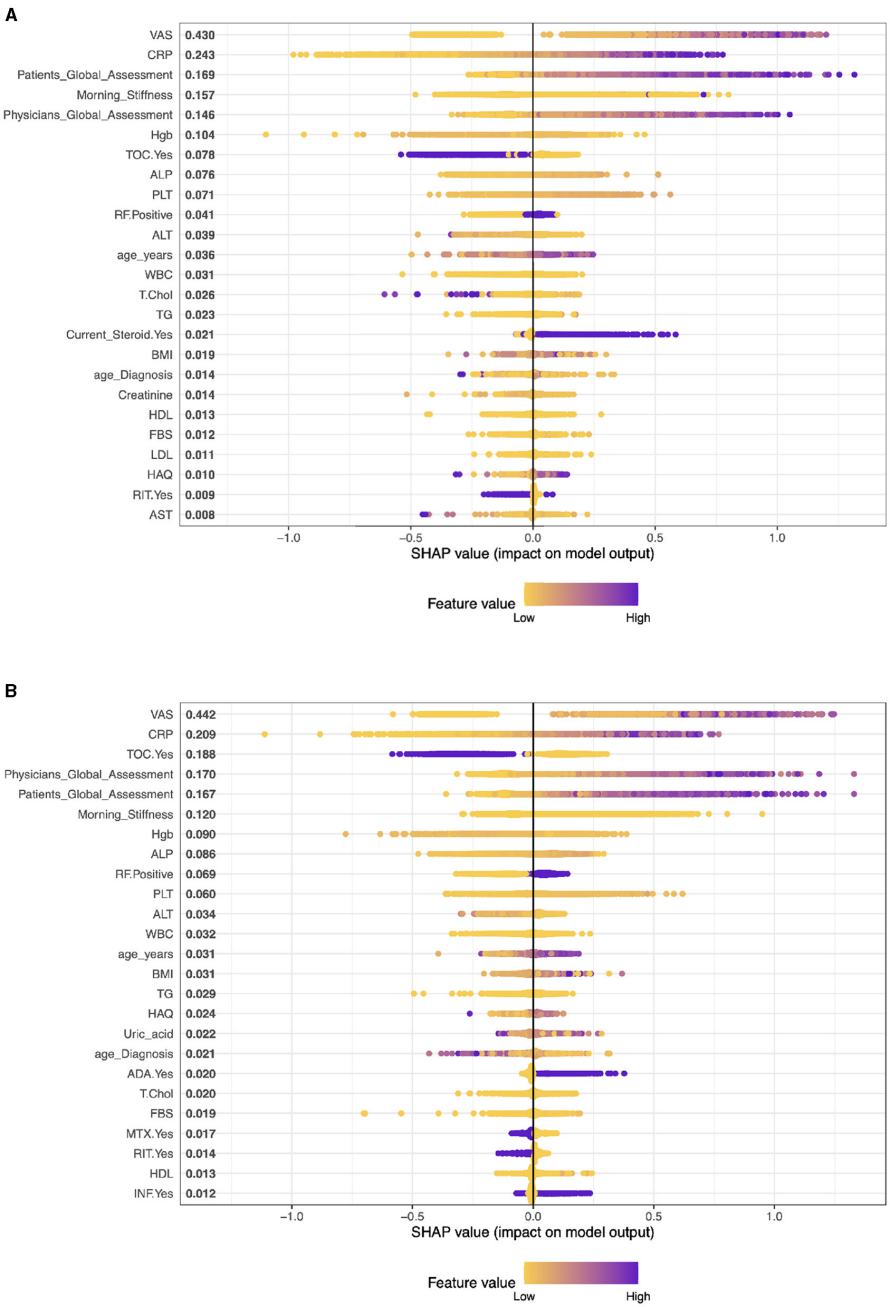
Importance matrix plot of the XGBoost model, depicting the importance of each factor for diagnosing RA pattern in SHAP summary plot for patients with cDMARDs and biologics. **(A)** Patients on cDMARDs. **(B)** Patients on biologics.

**Figure 2:**
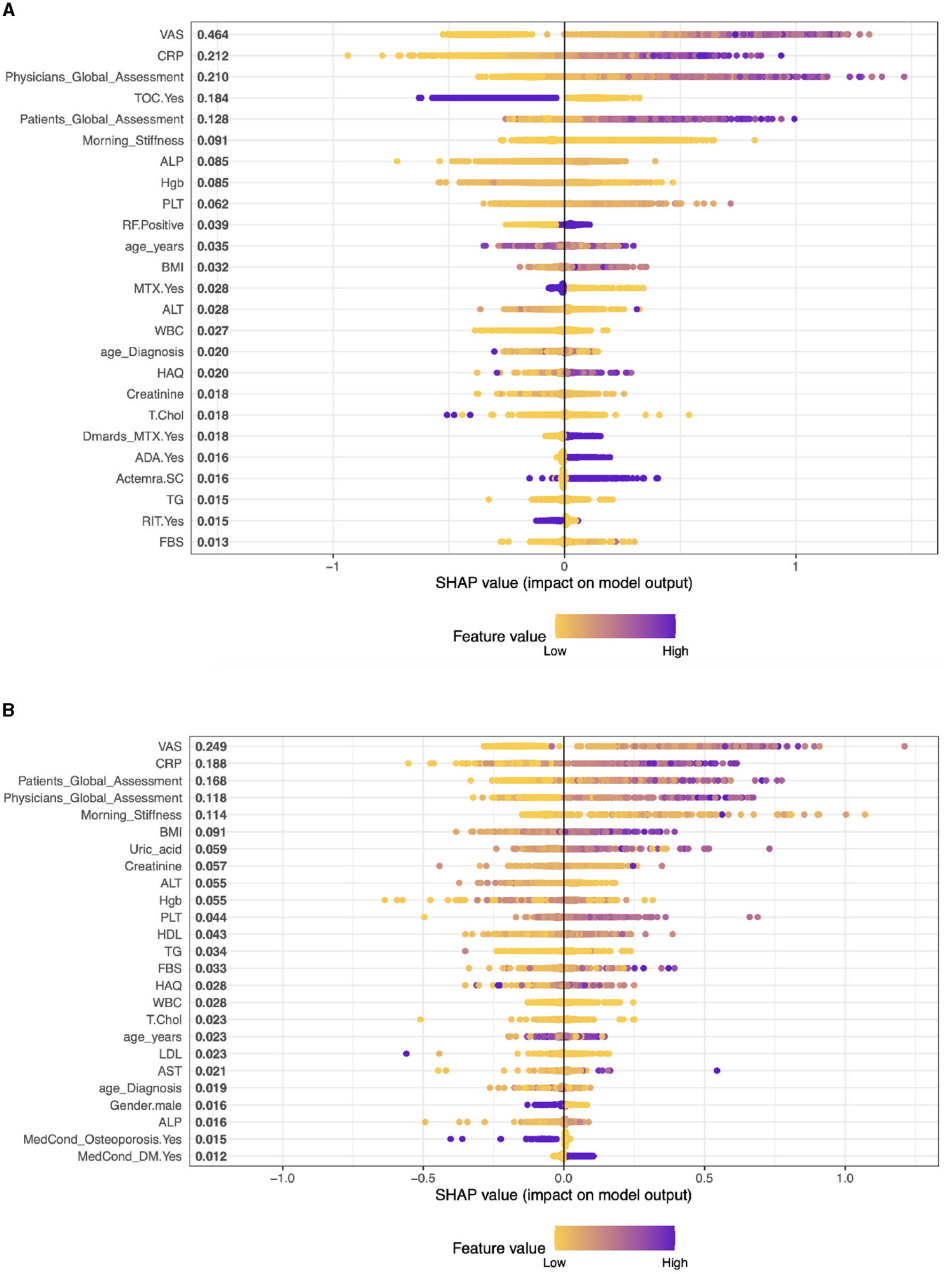
Importance matrix plot of the XGBoost model, depicting the importance of each factor for diagnosing RA pattern in SHAP summary for patients with biologics and cDMARDs (Combination) and Adalimumab treatment. **(A)** Biologics and cDMARDs (Combination). **(B)** Patients on Adalimumab drug.

**Figure 3:**
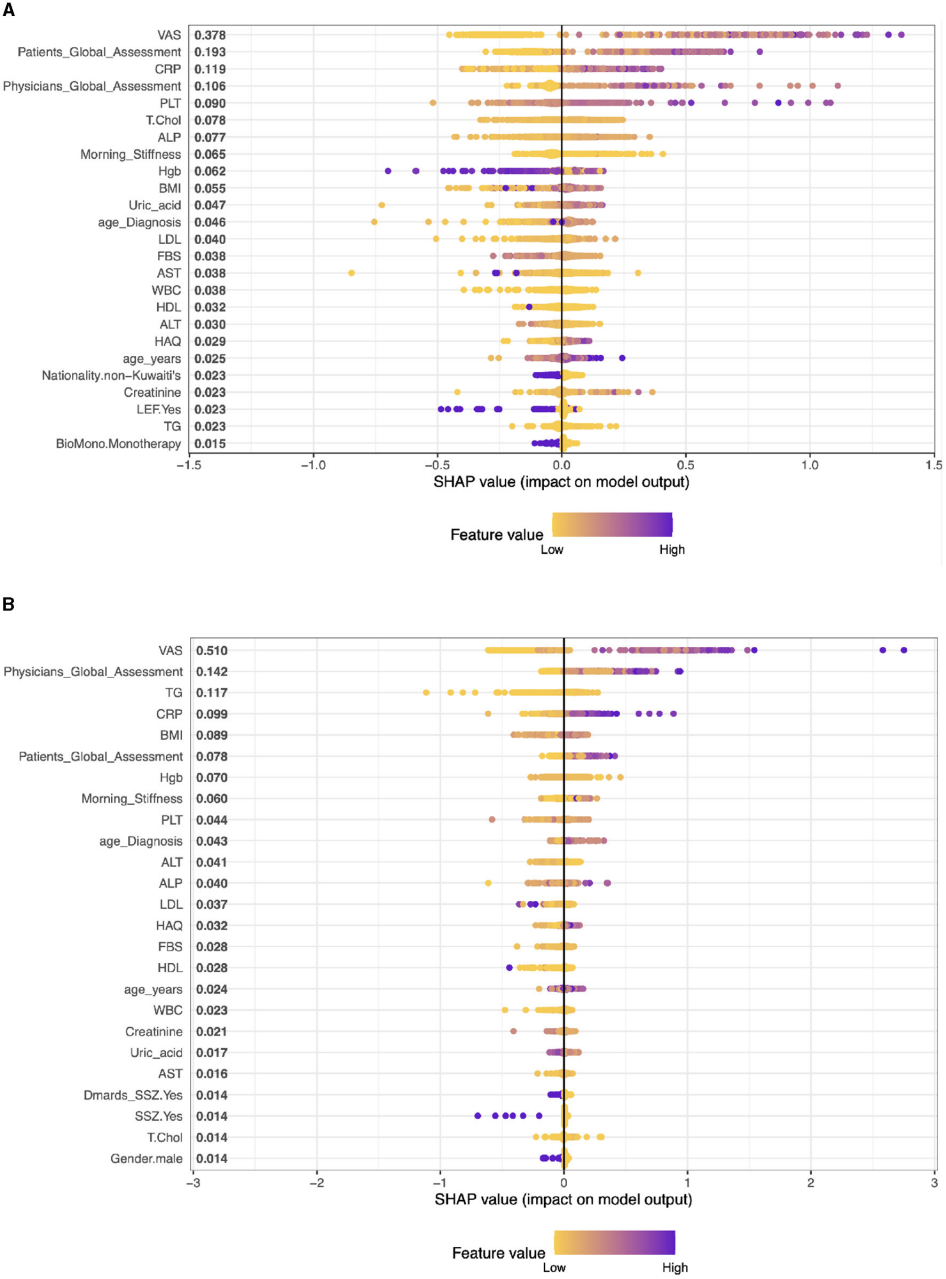
Importance matrix plot of the XGBoost model, depicting the importance of each factor for diagnosing RA pattern in SHAP summary plot for patients on Etanercept and Infliximab drug. **(A)** Patients on Etanercept drug. **(B)** Patients on Infliximab drug.

**Figure 4:**
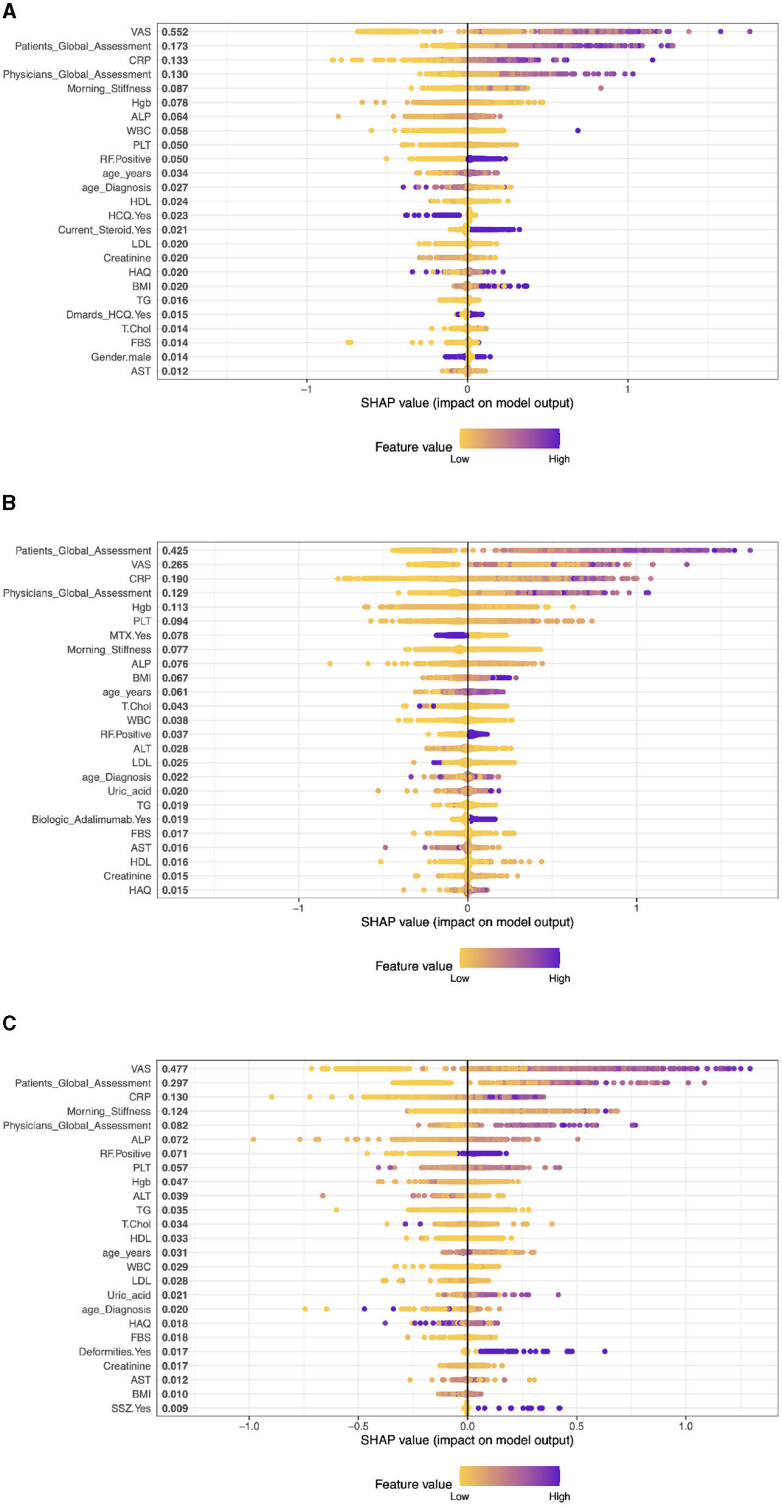
The XGBoost model's importance matrix plot shows the probability of DAS28 development for each factor in RA pattern diagnosis treated with Patients on Rituximab, Tocilizumab and Abatacept drug. **(A)** Patients on Rituximab drug. **(B)** Patients on Tocilizumab drug. **(C)** Patients on Abatacept drug.

Figures 1–**4** show SHAP scores for different types of patients treated. SHAP values measure the contribution of each feature to the prediction of each instance, averaged over all instances.

In this context (see Figure 1A), these SHAP values are for a model predicting some outcome for patients with Rheumatoid Arthritis (RA) who are undergoing conventional Disease-Modifying Anti-rheumatic Drug (cDMARD) treatment. The variables with larger SHAP values (VAS, CRP, Patient Global Assessment, etc.) have a more substantial impact on the model's prediction for each patient. The SHAP results suggest that VAS (Visual Analog Scale) is the most influential variable in the model with a SHAP value of 0.415. CRP has the second significant impact on the model's prediction with a SHAP value of 0.238. The Global Assessment of the patients is also very influential in the model (SHAP value: 0.174) with Morning Stiffness, which is another key determinant of the model output (SHAP value: 0.161).

#### 3.2.2 SHAP results for RA patients with biologics treatment

Figure 1B shows the list of mean SHAP values for various characteristics in a predictive model related to rheumatoid arthritis (RA) patients receiving biologics treatment (monotherapy). The values represent the average impact of each feature on the output of the model. As we mentioned earlier, a higher SHAP value indicates a higher average contribution to the model's prediction, either positively or negatively.

The analysis reveals that VAS has the highest level of influence on the prediction of the model, as indicated by its SHAP value of 0.414. This is followed by CRP with a SHAP value of 0.245, Physician's Global Assessment with a SHAP value of 0.210, Tocilizumab (TOC) patients with a SHAP value of 0.168, and Patient's Global Assessment with a SHAP value of 0.153. In contrast, the model shows that covariates such as HBV, HCV, certain DMARDs and biologics, and low Uric acid have negligible or no influence, as evidenced by their SHAP values close to zero.

#### 3.2.3 SHAP results for RA patients with biologics and cDMARDS treatment (combination)

In the context of interpreting SHAP values for patients with biologics and cDMARDS treatment (combination), Figure 2A shows that the importance of a characteristic is typically associated with the magnitude of the SHAP value.

In patients with RA who receive biologics and cDMARDs, the leading predictive features of the performance of DAS28, according to SHAP values, are VAS (0.438), Physician's Global Assessment (0.234), CRP (0.206), TOC (0.18), and morning stiffness (0.113). These values suggest the relative importance of each feature in influencing the response to treatment, with higher SHAP values indicating greater influence.

#### 3.2.4 Understanding the most important clinical features for RA patients with different biologics drugs

The application of machine learning to patient data holds significant promise in improving our understanding of disease progression and treatment efficacy in complex conditions such as Rheumatoid Arthritis (RA). In this analysis, we examine the contribution of various clinical features to the predictive models of RA patient outcomes, with a specific focus on patients receiving different biologic treatments. The key characteristics evaluated encompass both patient-reported outcomes, physician assessments, and laboratory markers indicative of disease activity.

**Case 1, Patients on Adalimumab drug (ADA):** The top five factors predicting outcomes in RA patients in the model are VAS (SHAP score: 0.281), CRP (0.170), Patient's Global Assessment (0.145), Physician's Global Assessment (0.135), and Morning Stiffness (0.092). These results highlight the significance of both subjective (patient and physician assessments) and objective measures (CRP, VAS, morning stiffness) in predicting treatment outcomes. Other clinical parameters like BMI, Creatinine, and ALT also contribute, albeit to a lesser extent, representing a mix of health indicators and laboratory results. (see Figure 2B).
**Case 2, Patients on Etanercept drug (ETA)**: The top five clinical features are: VAS With a SHAP score of approximately 0.375, Patients Global Assessment with a SHAP score of approximately 0.253, CRP, this feature has a SHAP score of approximately 0.169, PLT (Platelets) with a SHAP score of about 0.075, and Physicians Global Assessment with a SHAP score of approximately 0.071. The model also includes other factors like Morning Stiffness, T.Chol (Total Cholesterol), ALT (Alanine Aminotransferase), Hgb (Hemoglobin), and BMI (Body Mass Index) among others. These values represent a combination of clinical observations, laboratory values, and patient assessments (see Figure 3A).
**Case 3, Patients on Infliximab drug (INF)**: The top five clinical features in the model are VAS (SHAP 0.541), Physician's Global Assessment (0.146), BMI (0.109), TG (Triglycerides) (0.088), and CRP (0.082). These features, ranging from patient assessments to clinical and lab measures, play a significant role in predicting treatment outcomes, reflecting the interplay between physical symptoms, obesity, lipid profiles, and inflammation in RA (see Figure 3B).
**Case 4, Patients on Rituximab drug (RIT)**: The top five clinical features are: VAS with a SHAP value of approximately 0.557, then, Patients Global Assessment is the second most influential with a SHAP value of approximately 0.153. The third most important feature was Physicians Global Assessment with a SHAP value of about 0.147, then, CRP with a SHAP value of approximately 0.137. Morning Stiffness has the fifth most important feature with a SHAP value of about 0.083 (see Figure 4A).
**Case 5, Patients on Tocilizumab drug (TOC)**: In patients on Tocilizumab, the most influential clinical features are Patient's Global Assessment (SHAP 0.380), VAS (0.232), CRP (0.181), Physician's Global Assessment (0.178), and Morning Stiffness (0.112). These values indicate the importance of patient global and physician assessments, inflammation markers, and symptom severity in predicting disease activity and treatment response (see Figure 4B).
**Case 6, Patients on Abatacept drug (ABA)**: In RA patients on ABA, the key clinical features are VAS (SHAP 0.434), Patient's Global Assessment (0.359), CRP (0.132), Morning Stiffness (0.103), and Physician's Global Assessment (0.081). These elements highlight the impact of self-reported symptoms, inflammation levels, and physician evaluations in predicting disease activity and treatment responses (see Figure 4C).

#### 3.2.5 Machine learning models performance

**Figure 5:**
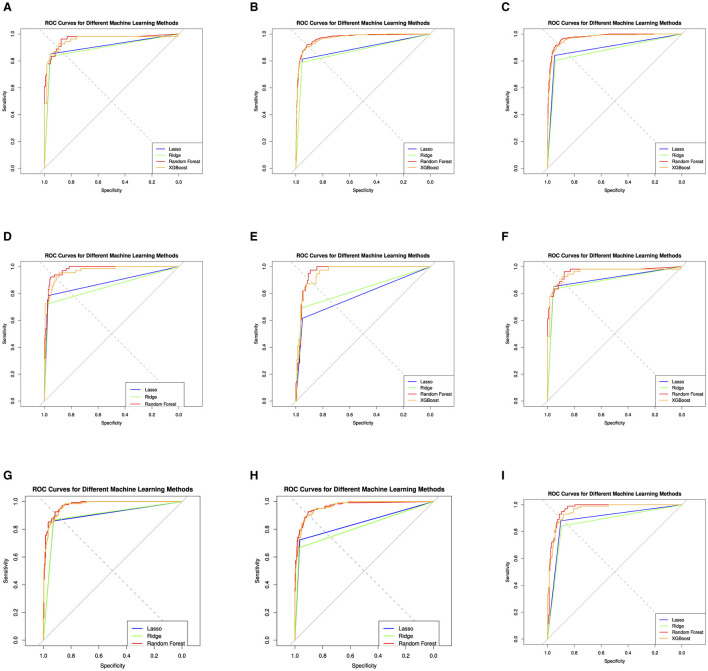
Evaluation of the four machine learning algorithms based on the AUC curve. AUC, area under the curve. The area under the receiver operating characteristic curves; Lasso Regression, Ridge Regression, Random Forest (RF), and XGBoost (extreme gradient boosting). **(A)** cDMARDs. **(B)** Monotherapy. **(C)** Combination. **(D)** Adalimumab. **(E)** Etanercept. **(F)** Infliximab. **(G)** Rituximab. **(H)** Tocilizumab. **(I)** Abatacept.

Table 3 and Figure 5 give the information on Sensitivity, Specificity, precision, and Accuracy that are used to evaluate the performance for several machine learning models including Support Vector Machine (SVM), Lasso Regression, Ridge Regression, Random Forest (RF), and XGBoost, for various treatments related to a medical context.

**Table 3:** Evaluating the performance of several machine learning models including Support Vector Machine (SVM), Lasso Regression, Ridge Regression, Random Forest (RF), and XGBoost, for various treatments.

**Treatment**	**Criteria**	**SVM**	**Lasso**	**Ridge**	**RF**	**XGBoost**
cDMARDs	Sensitivity	0.946	0.944	0.946	0.955	0.955
	Specificity	0.806	0.830	0.789	0.860	0.852
	Precision	0.923	0.932	0.917	0.944	0.941
	Accuracy	0.906	0.911	0.900	0.927	0.925
Biologics	Sensitivity	0.946	0.947	0.955	0.953	0.948
	Specificity	0.814	0.831	0.807	0.868	0.852
	Precision	0.935	0.940	0.933	0.953	0.947
	Accuracy	0.912	0.917	0.916	0.931	0.923
Combination	Sensitivity	0.956	0.962	0.958	0.959	0.952
	Specificity	0.814	0.840	0.809	0.851	0.856
	Precision	0.934	0.944	0.933	0.947	0.948
	Accuracy	0.918	0.930	0.918	0.930	0.927
ADA	Sensitivity	0.960	0.965	0.971	0.971	0.965
	Specificity	0.631	0.754	0.646	0.846	0.800
	Precision	0.874	0.913	0.880	0.944	0.928
	Accuracy	0.870	0.908	0.882	0.937	0.920
ETA	Sensitivity	0.929	0.943	0.943	0.943	0.936
	Specificity	0.769	0.769	0.769	0.872	0.769
	Precision	0.936	0.937	0.937	0.964	0.936
	Accuracy	0.894	0.906	0.906	0.928	0.900
INF	Sensitivity	0.902	0.964	0.973	0.973	0.955
	Specificity	0.463	0.870	0.833	0.889	0.852
	Precision	0.777	0.939	0.924	0.948	0.930
	Accuracy	0.759	0.934	0.928	0.946	0.922
RIT	Sensitivity	0.909	0.938	0.931	0.925	0.934
	Specificity	0.875	0.890	0.846	0.897	0.890
	Precision	0.945	0.952	0.934	0.955	0.952
	Accuracy	0.899	0.923	0.906	0.917	0.921
TOC	Sensitivity	0.968	0.963	0.974	0.970	0.967
	Specificity	0.667	0.732	0.699	0.756	0.764
	Precision	0.931	0.943	0.938	0.949	0.950
	Accuracy	0.915	0.922	0.925	0.932	0.931
ABA	Sensitivity	0.857	0.888	0.882	0.901	0.907
	Specificity	0.861	0.851	0.832	0.931	0.881
	Precision	0.908	0.905	0.893	0.954	0.924
	Accuracy	0.859	0.874	0.863	0.912	0.897

The performance of these models is also assessed using a Receiver Operating Characteristics (ROC) curve, a common method to evaluate the quality of machine learning models in medical applications. For the cDMARDs treatment, the Random Forest and Xgboost models perform the best overall, with the highest sensitivity (0.955), and high values for specificity, precision, and accuracy. For biologics treatment, the Random Forest model performs the best, with the highest values in three categories: sensitivity, specificity, and precision. In the Combination treatment category, the Lasso and Random Forest models are tied for the best performance with the highest accuracy (0.930). For the ADA treatment, the Random Forest model outperforms the others with the highest values in three categories: sensitivity, specificity, and accuracy. For the ETA and TOC treatments, the Random Forest model again shows the highest performance in most categories. In case of INF treatment, the Random Forest model performs best in terms of specificity, precision, and accuracy, while the SVM model has the lowest accuracy. For RIT treatment, Lasso and Xgboost models tie for the highest accuracy, while Random Forest has the highest precision and specificity. In ABA treatment, Random Forest again stands out as the best model in terms of specificity, precision, and accuracy. In summary, overall, the RF and XGBoost models generally outperform SVM, Lasso, and Ridge in the different treatments. RF usually performs slightly better than XGBoost, particularly in terms of Specificity and Precision.

## 4 Discussion

### 4.1 Machine learning methods in RA

In this study, we employed various machine learning techniques to predict remission in rheumatoid arthritis (RA) patients treated with biologic disease-modifying anti-rheumatic drugs (bDMARDs). By integrating Support Vector Machine (SVM), Lasso and Ridge regression, Random Forest, XGBoost, and Shapley Additive Explanation (SHAP), we leveraged the strengths of each method to enhance predictive accuracy and interpretability. SVM was effective for classification tasks, particularly in high-dimensional spaces, while Lasso and Ridge Regression addressed overfitting and multicollinearity. Random Forest, with its ensemble approach, provided robust predictions by aggregating multiple decision trees. XGBoost offered high performance and efficiency in identifying significant clinical features related to treatment responses, and SHAP values provided clear insights into the contribution of each feature to the model's predictions. The consolidation of these machine learning methods allowed us to identify key clinical characteristics that influence RA remission, such as the Visual Analog Scale (VAS), C-reactive protein (CRP), patient global assessment, and physician global assessment. These findings underscore the importance of a multifaceted approach in predictive modeling, combining various techniques to achieve a comprehensive understanding of treatment outcomes.

### 4.2 Selecting the most clinical features using SHAP

In our study, we started with the SHAP approach to select the most influential factors that control DAS28 for different types of RA patients in Kuwait. The purpose of the Shapley Additive Explanations (SHAP) approach is to provide explanations of machine learning models and to evaluate the importance of variables or features in predictive models (Fan, 2023; Koo et al., 2022). SHAP allows for the interpretation of the contributions of different features to the model's predictions (Li et al., 2022). The SHAP approach can be used to classify the importance of particle properties in determining the degree of particle damage in battery cathodes (Li et al., 2022). It has also been applied to evaluate variable importance and temporal importance in LSTM models (Fan, 2023), interpret tree-based models to predict rent in real estate (Lenaers et al., 2023), explain the output of event machine learning classifiers (Pezoa et al., 2023), detect chronic heart disease (Admassu, 2023), interpret phase-resolved partial discharge signals (Kitani and Iwata, 2023), and analyze various agricultural worker data (Kawakura et al., 2022). Furthermore, SHAP can be used to forecast patient outcomes in kidney exchange and to provide consistent explanations for variation in match outcomes (Hu et al., 2022).

### 4.3 The most important clinical features among RA patients from KRRD

Our study demonstrates the most clinical features for different types of RA patients from KRRD. In this study, we split the patients into nine different groups based on the treatments received as we mentioned before. The first group includes all RA patients on cDMARD. Our SHAP results show that the most important clinical characteristics that control DAS28 are VAS, CRP, Global Assessment of Patients, and Morning Stiffness, and this is agreed by Sadura-Sieklucka et al. (2019), Sas (2019), and Mohammed et al. (2013).

These clinical features, including VAS, CRP, PGA, and morning stiffness, are valuable in assessing disease activity and guiding treatment decisions in RA patients with cDMARD. The DAS28, which incorporates these characteristics, is widely used in clinical practice and research (Asmussen et al., 2013; Inoue et al., 2007). Obtaining control of these clinical features can help optimize disease management and improve patient outcomes.

For the second group (RA patients on biologics), SHAP results confirmed the most clinical characteristics that control DAS28. VAS was the most influential factor along with CRP, Physicians' Global Assessment, Tocilizumab (TOC) and Patient's Global Assessment. Factors that were found to be associated with disease activity in patients with rheumatoid arthritis (RA) according to the Disease Activity Score 28 (DAS28) include age, level of erythrocyte sedimentation rate (ESR), visual analog scale of pain (VAS), and C-reactive protein (CRP) (Kurniari and Kambayana, 2021; Abdelnaby et al., 2021; Al-Shamali et al., 2021). In addition, DAS28-CRP was found to have an association with fatigue measured using VAS (Doumen et al., 2022). Furthermore, DAS28 scores were found to have the highest divergence in patients with discordant assessments of global disease severity (Barton et al., 2010).

Based on machine learning random forest analysis, the 28-year RA disease activity score (RA DAS28) based on the Visual Analog Scale (VAS) was determined to be the most influential factor in assessing disease activity in patients with rheumatoid arthritis (RA) (Cordingley et al., 2014; Kurniari and Kambayana, 2021; Yoshii et al., 2018). This finding is supported by studies that have found a strong correlation between RA DAS28 VAS and patient-reported outcomes, such as pain and overall disease activity (Kim et al., 2016).

Furthermore, machine learning models have been developed to predict remission in RA patients treated with biologic disease-modifying antirheumatic drugs (bDMARDs) using readily available demographic and clinical factors (Alsaber, 2023). The Kuwait Registry for Rheumatic Diseases was used to collect follow-up data from patients with RA treated with bDMARD, and the model predicted remission using baseline clinical data. Machine learning approaches have also been used to predict responses to methotrexate treatment in RA patients. Athreya et al. (2022) applied machine learning with clinical and genomic biomarkers to predict response to methotrexate treatment in patients with early RA. They used demographic, clinical, and genomic data from the cohort and found that machine learning approaches could predict the response to treatment. Several other studies have used machine learning to predict various aspects of disease activity, response to treatment, and patient outcomes in RA. For example, Shipa et al. (2021) investigated the synergistic efficacy of hydroxychloroquine with methotrexate using machine learning analysis. Lee et al. (2021) developed prediction models for responses of biologic disease-modifying antirheumatic drugs (bDMARDs) in patients with RA and ankylosing spondylitis using machine learning. Jenko et al. (2018) constructed a clinical pharmacogenetic index using machine learning to predict responses to methotrexate monotherapy in Slovenian and Serbian RA patients. In general, these studies highlight the utility of machine learning approaches in predicting the clinical characteristics of DAS28 in RA patients. Machine learning models have demonstrated the ability to identify important predictors of disease activity, treatment response, and outcomes in RA, providing valuable insights for personalized medicine and optimizing patient care.

### 4.4 Limitation and future studies

One difficulty of machine learning in medical data analysis is the lack of standards in data representation and model benchmarking. It can be challenging to evaluate and validate machine learning models across datasets due to the wide variety of data formats and standards used by various hospital systems and health care. Another limitation is that machine learning algorithms themselves can have an effect on their efficacy when analyzing medical data. Challenges in data access and integration, data privacy and confidentiality, lack of standardization, limitations of machine learning algorithms, and limited availability of open access data are among the limitations for medical data analysis using machine learning. To effectively utilize the potential of machine learning to improve medical diagnosis, treatment decision making, and patient care, these limitations must be overcome. The Kuwait Registry for Rheumatic Diseases (KRRD) offers a comprehensive database; however, the precision and completeness of the data is based on the meticulousness of clinical record keeping methods. The precision of the information retrieved can be affected by variability in documentation standards, the potential for human error in data entry, and the occasional incompleteness of the records. This constraint is especially significant when working with extensive datasets over prolonged durations, as even slight discrepancies or omissions in the data can have a cumulative effect on overall conclusions. We have made significant efforts to reduce these problems by using thorough data validation and quality control procedures. However, it is crucial for the reader to take into account this possible influence of bias while analyzing the findings of the study.

We recommend that for future studies, researchers demonstrate the potential of machine learning in predicting disease activity scores and treatment responses in patients with RA using additional features such as physical activity data, genetic markers, and patient-reported scales.

## 5 Conclusion

In conclusion, our study successfully developed machine learning models using data from the Kuwait Registry of Rheumatic Diseases (KRRD) to predict remission in patients with rheumatoid arthritis (RA) treated with different biologic disease-modifying antirheumatic drugs (bDMARDs). Using various machine learning techniques, including lasso, ridge, support vector machine, random forest, XGBoost, and Shapley additive explanation (SHAP), we were able to determine the key clinical variables associated with remission for each biologic.

Our findings revealed that certain factors exhibited a stronger association with remission than others, although the classification of these factors varied between different biologics. Specifically, age was the most important feature for adalimumab, rheumatoid factor for etanercept, erythrocyte sedimentation rate for infliximab and golimumab, duration of disease for abatacept and C-reactive protein for tocilizumab. These insights were obtained through the utilization of the Shapley plot, a component of explainable artificial intelligence (XAI), which provided valuable information on the effects of predictors on the prognosis of remission for each bDMARD. Using our methodology, clinicians can obtain valuable information on important clinical information associated with remissions, potentially enhancing the efficacy of treatment for individuals affected by rheumatoid arthritis.

In summary, our study highlights the effectiveness of machine learning models in predicting remission outcomes in RA patients treated with different bDMARDs. Identifying clinical characteristics that show predictability of remission for each biologic can significantly contribute to personalized treatment approaches and improve patient outcomes in the management of rheumatoid arthritis.

## Data Availability

The original contributions presented in the study are included in the article/supplementary material, further inquiries can be directed to the corresponding author.
